# Experimental validation of a spectroscopic Monte Carlo light transport simulation technique and Raman scattering depth sensing analysis in biological tissue

**DOI:** 10.1117/1.JBO.25.10.105002

**Published:** 2020-10-27

**Authors:** Alireza Akbarzadeh, Ehsan Edjlali, Guillaume Sheehy, Juliette Selb, Rajeev Agarwal, Jessie Weber, Frédéric Leblond

**Affiliations:** a Polytechnique Montréal, Department of Engineering Physics, Montreal, Quebec, Canada; b Centre de Recherche du Centre Hospitalier de l’Université de Montréal, Montreal, Quebec, Canada; c ODS Medical Inc., Montreal, Quebec, Canada; d Institut National d’Optique, Quebec, Quebec, Canada

**Keywords:** Raman spectroscopy, elastic scattering, fluorescence, Monte Carlo simulation, tissue optics, metrology

## Abstract

**Significance:** Raman spectroscopy (RS) applied to surgical guidance is attracting attention among scientists in biomedical optics. Offering a computational platform for studying depth-resolved RS and probing molecular specificity of different tissue layers is of crucial importance to increase the precision of these techniques and facilitate their clinical adoption.

**Aim:** The aim of this work was to present a rigorous analysis of inelastic scattering depth sampling and elucidate the relationship between sensing depth of the Raman effect and optical properties of the tissue under interrogation.

**Approach:** A new Monte Carlo (MC) package was developed to simulate absorption, fluorescence, elastic, and inelastic scattering of light in tissue. The validity of the MC algorithm was demonstrated by comparison with experimental Raman spectra in phantoms of known optical properties using nylon and polydimethylsiloxane as Raman-active compounds. A series of MC simulations were performed to study the effects of optical properties on Raman sensing depth for an imaging geometry consistent with single-point detection using a handheld fiber optics probe system.

**Results:** The MC code was used to estimate the Raman sensing depth of a handheld fiber optics system. For absorption and reduced scattering coefficients of 0.001 and $1\;{\mathrm{mm}}^{-1}$, the sensing depth varied from 105 to 225  μm for a range of Raman probabilities from $10^{-6}$ to $10^{-3}$. Further, for a realistic Raman probability of $10^{-6}$, the sensing depth ranged between 10 and 600  μm for the range of absorption coefficients 0.001 to $1.4\;{\mathrm{mm}}^{-1}$ and reduced scattering coefficients of 0.5 to $30\;{\mathrm{mm}}^{-1}$.

**Conclusions:** A spectroscopic MC light transport simulation platform was developed and validated against experimental measurements in tissue phantoms and used to predict depth sensing in tissue. It is hoped that the current package and reported results provide the research community with an effective simulating tool to improve the development of clinical applications of RS.

## 1 Introduction

Raman spectroscopy (RS) has been under considerable investigation in biomedical optics during the last several decades.^1^^–^^7^ Compared to more established biomedical probing and imaging techniques [e.g., fluorescence imaging, magnetic resonance imaging (MRI), computed tomography (CT), nuclear imaging], RS offers several advantages. It relies on nonionizing radiation; it can be integrated into standard medical workflows because of its potential integration into compact fiber optics systems; it can be either label-free or applied to image-specific targeted ligands; and furthermore, it offers easy sample preparation and compatibility with aqueous solvents. However, there are two major drawbacks to the application of label-free RS in biomedical optics. Because of its low conversion rate, the Raman scattering cross section is weak compared to other light–tissue interaction mechanisms, which makes Raman imaging a slow process. Moreover, Raman scattering is more likely to occur in the Stokes regime where photons are redshifted, which makes the Raman photon detection process prone to contamination by potentially large fluorescence background from native biomolecules in tissue and cells.

Practical clinical applications of RS for tissue imaging (e.g., surgical guidance, targeted biopsy collection, and treatment monitoring) were mostly developed using fiber optics systems without regard to controlling tissue sensing depth. As a result, these approaches can be inherently imprecise when used for surgical guidance or targeted biopsy collection. Moreover, ambiguities in tissue sampling depth make the whole process prone to modeling errors when using pathology analysis to train predictive models based on supervised machine learning. Therefore, deriving a rigorous relation between the Raman sensing depth for specific imaging systems and tissue optical properties (absorption and elastic scattering) will be essential for the development of real-world RS applications in medicine. More recently, advances were made in depth-resolved techniques and developing spatial-offset Raman spectroscopy (SORS) with various types of probes and optical setups to harvest Raman signals at depth.^8^^–^^16^ These innovative techniques would also greatly benefit from robust simulation tools for modeling the depth sensitivity of RS.

Attaining a rigorous relation between Raman depth and optical properties in an RS system requires solving the radiative transfer equation (RTE) and calculating photon diffusion with predetermined boundary conditions. Light transport simulations in tissue optics traditionally involve modeling three competing interaction mechanisms: absorption and photon energy conversion into heat (resonant phenomenon), fluorescence (resonant, but redshifted re-emission), and elastic scattering (nonresonant).^17^^,^^18^ These phenomena can be modeled by analytically or numerically solving the RTE; this can be simplified to the diffusion equation ($P_{1}$ approximation) or to the simplified spherical harmonics equation ($SP_{N}$ approximation) in highly scattering media. However, these analytical/numerical approaches are limited when modeling complex imaging domains with realistic heterogeneities or curved geometries.

Biological tissue can be approximated as a random medium in which light is mainly diffused by going through many scattering incidents. This problem is either analytically impossible to solve for realistic imaging configurations or it can be computationally intensive in numerical simulations to account for absorption, elastic scattering, and fluorescence. An alternative to the analytical/numerical studies is the Monte Carlo (MC) method, which was proposed for the first time by Wilson and Adam^19^ and improved by several groups^20^^–^^22^ to solve the problem of light transport in tissue. Due to its stochastic nature, MC is essentially capable of dealing with any level of complexity concerning optical or geometrical properties in the RTE, as long as appropriate computational platform (hardware and software) is provided. The advent of strong microprocessors boosted with modern graphical processing units (GPU) accompanied by robust parallelization algorithms in recent years have paved the road for MC to produce rigorous and reliable solutions to light transport in tissue. In MC simulations, photons undergo sequential random walks and, during each step, the corresponding optical parameters (absorption, elastic scattering, fluorescence, and Raman scattering) can be randomly sampled to decide which of the four competing events happens along the walk.

In this article, a new MC package is introduced that can simulate elastic and inelastic scattering of photons in biological tissue. First, a review of the previously reported work on MC analysis of photon transport is provided and the advantages of the package presented in this article are highlighted. Then, the stochastic analysis methodology of elastic and inelastic diffusion of photons is presented. To validate the new package, simulation results in realistic tissue phantoms are presented and compared with those of experimental phantoms of known absorption and scattering coefficients. Detailed Raman sampling depth analysis is then presented to establish relationships between sensing depth, tissue optical properties, and imaging geometry parameters. This work sets the stage for the development of more controlled experimental protocols leading to the clinical translation of RS systems into medical applications.

## 2 Monte Carlo Simulation Strategy

### 2.1 Overview of MC Simulators in Biomedical Optics

Various MC algorithms have been implemented to stochastically solve the RTE and study light transport in turbid media, including biological tissue. Monte Carlo Multi-Layered (MCML) was the first MC package developed by Wang et al.^23^ in standard C language to model photon diffusion in layered tissue. MCML initially had two limitations: the first was its long computational time, which could take many hours for a single simulation, and the second was its restriction to model only layered geometries and its inability to simulate nonlayered structures. Concurrent with MCML, Wang et al.^24^ released convolutional MCML (CONV MCML) to simulate the interaction between a finite-width light beam and multilayered tissues. The advent of more powerful computational methods as well as the development of multicanonical MC simulators^25^ helped to overcome the first drawback of MCML. Hybrid models were also introduced to model light transport in more complex geometries, allowing inclusion of simple geometrical shapes such as cuboids, spheres, and cylinders in layered structures.^26^^,^^27^ Among the reported results obtained from the hybrid models were MC study of optimized light delivery for tumor laser treatment,^28^ MC modeling of light delivery and focusing in tissue with blood vessels, arteries, and capillaries,^29^^–^^31^ MC analysis of photoacoustic imaging of sentinel lymph nodes,^32^ MC study of near-infrared (NIR) light propagation within adult and neonatal head models,^33^ and grid-based modeling of skin under laser irradiation.^34^ In recent decades, the hybrid models were superseded by more versatile techniques such as CUDAMCML,^35^ mesh-based MC,^36^ MC eXtreme,^37^ tetrahedron-based inhomogeneous MC optical simulator,^38^ and voxel-based MC.^39^

In addition to the traditional MC simulation techniques, where mainly elastic scattering and absorption of light in tissue are modeled, a substantial number of MC studies investigating fluorescence^40^^–^^51^ and the Raman effect^52^^–^^62^ in turbid media have been reported. Modification of the primary MC approaches incorporating the quantum yield and emission spectra of specific fluorophores were used to stochastically model fluorescence in scattering and absorbing media.^40^^–^^45^ In two consecutive papers,^46^^,^^47^ MC simulations were applied to empirically model the fluence rate and fluorescence re-emission as a function of effective penetration depth and diffuse reflectance. Beuthan et al.^48^ showed that $C_{21}H_{27}N_{7}O_{14}P_{2}$ (NADH) concentration in turbid media can be estimated through simultaneous detection of fluorescence and light backscattering. This work was extended by Minet et al.^49^ to estimate the concentration of NIR fluorophores. In another work, a fast Fourier method and scaled fitting procedures were used to improve the speed and accuracy of the MC simulations in reconstructing the fluorescence spectra.^50^^,^^51^

A deficiency of existing MC tools is that they are unable to simultaneously account for all tissue optics competing mechanisms, including not only absorption/elastic scattering and fluorescence, but also nonelastic scattering, i.e., the Raman effect. Enejder et al.^52^ used an MC method to model the Raman-scattered light within blood samples in a quartz cuvette and quantified the analytes in whole blood. A semi-analytical study facilitated by an MC simulation on infinite and finite single-layer media to correct the turbidity-induced distortions in Raman spectra was presented by Shih et al.^53^. Mo et al.^54^ presented an optical fiber Raman probe coupled with a ball lens and developed an MC simulation to study the depth-resolved Raman signal collected from a two-layer epithelial tissue. Hokr and Yakovlev^55^ developed an MC model to consider elastic scattering, absorption, and spontaneous Raman scattering in a turbid medium and they showed that an enhancement in elastic scattering leads to the growth of forward and backward Raman signals. However, due to the extensive computational costs of the proposed MC model, the authors chose artificially large values (on the order of $10^{-2}$) for the effective Raman cross section and made the assumption that the laser pump depletion is negligible, which may hinder the given conclusion in realistic inelastic scattering light–tissue interaction measurements. An MC approach for calculating the depth sensitivity of single-fiber and multi-fiber Raman probes interrogating skin was presented by Reble et al.^56^. Modeling skin as a two-layer geometry with a finite epidermis layer backed by a semi-infinite dermis layer, the authors demonstrated that skin with nonmelanoma cancer reveals higher sampling depth compared to normal skin. In 2014, two groups separately developed MC simulations with Raman modeling capabilities. Wang et al.^57^ built an eight-layer skin model and performed an MC calculation to study how different layers affect the measured *in vivo* Raman spectrum. Hokr et al.^58^ presented a model based on the MCML package and investigated the stimulated Raman scattering and nonlinear dynamics of light in turbid media. Both the proposed models were, however, limited to layered structures, which limits their applications for more complex geometries. Periyasamy et al.^59^^,^^60^ presented Raman MC simulations based on the MCML package with embedded objects with spherical, cuboidal, ellipsoidal, and cylindrical shapes in layered structures. MC calculations were also conducted to analyze SORS systems.^16^^,^^61^^,^^62^

All the cited studies on the use of MC methods to analyze Raman scattering are either limited to layered structures with simple embedded shapes or suffer from a high computational cost in simulating Raman photons. The work presented here overcomes those difficulties by enabling rapid GPU-boosted simulation of all four competing mechanisms, including the Raman phenomenon, in turbid tissues with any arbitrary geometry or optical properties over any desired spectral range. The availability of this tool is important as it will highlight the roadmap toward the fabrication of more realistic optical phantoms, determine the Raman depth sensitivity across all possible tissue parameters, and make a quantitative assessment of expected levels of Raman signal-to-noise ratio (SNR), which is dramatically impacted by the relatively high levels of fluorescence and heterogeneity of biological tissues.

### 2.2 New MC Simulator and Its Advantages Over Currently Available Methods

In this section, a newly developed MC package is presented for simulating elastic and inelastic tissue light scattering. The current MC package was developed based on parallelization of the algorithm on graphics cards, which is obtained using the Open Graphics Library (OpenGL).^63^ Compared to other published Raman simulators, the developed package offers several important features that are emphasized here.

First, a marching cube algorithm facilitated by several shaders from the OpenGL shading language was used in the implementation of the medium that allows robust rendering of three-dimensional (3-D) curved geometries with locally refined structures that may contain multiple inclusions of different optical properties. In generating the geometries, the voxelated 3-D objects are saved in stack of two-dimensional (2-D) images and the marching cube algorithm performs the divide-and-conquer approach to extract isosurfaces (3-D regions with identical optical properties). More details on the applied marching cube algorithm can be found in Refs. 63 and 64. Second, the package was developed essentially by incorporating several shaders of OpenGL, including vertex shaders, fragment shaders, and geometry shaders. By means of these shaders, all the MC calculations regarding light transport, processing the initial and final positions, directions, and wavelengths of photons, reflection and refraction of photons, geometry rendering, as well as camera/sensor implementation are performed on the GPU. Consequently, the running time of each simulation is shorter compared to CPU-based packages. Furthermore, due to the flexibility of OpenGL and its compatibility with many graphic cards, the developed package does not require a very specific architecture and can be run on any computer with a graphic processor supporting OpenGL 4.1 or above. Thus, significant benefit in portability, implementation, and maintenance of the package is brought forth. Third, the package parallel implementation is robust and dynamic such that the wavelength shift of photons, due to inelastic scattering and fluorescence events, and their diffusion at the shifted wavelengths can be simulated concurrently with the propagation of photons at the launching wavelength. Therefore, the MC package is capable of simulating both fluorescence and Raman spectra, i.e., phenomena at multiple wavelengths. Fourth, the demonstrated package offers easy access to information regarding the final location and direction of photons, their final wavelengths, as well as the locations where inelastic scattering, fluorescence, or absorption events occurred. Having access to this information provides users with the ability to perform various types of post-processing analysis, such as spatial or temporal filtering. Fifth, the package allows for inputs from structural imaging modalities such as MR and CT, which allows simulations in realistic situations through different organs and within contrast-enhancing tumors. Sixth, the MC simulator provides the user with several light source and sensor options. For illumination, different types of sources such as point source, wide field source with the Gaussian profile, and patterned illumination for spatial frequency-domain imaging can be modeled. The detection features include single-point fiber optics detection and wide-field camera-based detection.

### 2.3 Stochastic Modeling of Light–Tissue Interaction and Random Variables

In a turbid medium, photons are traveling random paths composed of a sequence of straight segments. Each path can be interrupted by one of the following four phenomena: elastic scattering (Rayleigh or Mie), absorption, fluorescence, or inelastic (Raman) scattering. To include each of these events in the MC analysis, their respective phenomenological physical constants are used to build a probability density function (PDF) and hence, sample random numbers. For elastic scattering, the scattering coefficient $\mu_{s}$ represents the scattering probability per unit distance along each photon trajectory. Based on a modified version of the Beer–Lambert law, the PDF and cumulative distribution function (CDF) for elastic scattering are defined as follows:^18^

$$f_{s}(x,\mu_{s})=\mu_{s}e^{-\mu_{s}x},$$ (1)

$$F_{s}(l,\mu_{s})=\overset{l}{\underset{0}{\int }}\mu_{s}e^{-\mu_{s}x}dx=1-\mu_{s}e^{-\mu_{s}l}.$$ (2)

The function $F_{s}$ is the CDF, which represents the probability that a photon has gone through a scattering event after having traveled a distance l. Then for the length of the photon path between two diffusion events ($l_{\mathrm{diff}}$), a random number $\xi_{1}$, between 0 and 1, is generated and following the elastic scattering CDF, we have

$$\xi_{1}=1-\mu_{s}e^{-\mu_{s}l_{\mathrm{diff}}},$$ (3)

which gives

$$l_{\mathrm{diff}}=-\frac{1}{\mu_{s}}\;\ln(\frac{1-\xi_{1}}{\mu_{s}}).$$ (4)

In addition to the diffusion length, the direction of elastic scattering is determined from the phase function, which is essentially the angular probability density of a photon coming from solid angle direction Ω being scattered into direction $\Omega^{\prime }$, i.e., $p(\Omega ,\Omega^{\prime })$. For the case of unpolarized light, the probability of scattering is equally distributed for all the angles in the azimuthal plane, i.e., $p_{\phi }(\phi )=\phi /2\pi$ and hence $p(\Omega ,\Omega^{\prime })=p(\Omega \cdot \Omega^{\prime })=p(\cos\;\theta )$. To determine the azimuthal direction of the scattering, a random number $\xi_{2}$, distributed uniformly between 0 and 1, is generated and the azimuthal direction of scattering is obtained as $\phi =2\pi \xi_{2}$. Then, by assigning the Henyey–Greenstein phase function^17^^,^^18^ to p(cos θ), the CDF of the scattering angle in the polar plane is

$$P_{\theta }(\cos\;\theta )=\frac{1}{2}\overset{\cos\;\theta }{\underset{-1}{\int }}p(\cos\;\theta )d(\cos\;\theta ).$$ (5)

By letting $P_{\theta }(\theta )=\xi_{3}$, where $\xi_{3}$ is distributed between 0 and 1, the scattering orientation along the polar plane is, thus,

$$\theta =\arccos\{\frac{1}{2g}[1+g^{2}-{\left(1-\frac{g^{2}}{1-g+2g\xi_{3}}\right)}^{2}]\},$$ (6)

where g is the anisotropy coefficient.

The absorption coefficient $\mu_{a}$ specifies the probability of absorption per unit distance traveled by a photon with corresponding CDF,

$$F_{a}(l,\mu_{a})=\overset{l}{\underset{0}{\int }}\mu_{a}e^{-\mu_{a}x}dx=1-\mu_{a}e^{-\mu_{a}l},$$ (7)

which indicates the probability that an absorption event occurs within a traveled distance l. Some MC approaches maintain a packet weight index for each photon, which is decreased by absorption during the photon travel.^18^ Here, the “intensity” of the photons is unaltered along their paths and they are considered as quantized (albeit nonpolarized) particles. Absorption is considered on the same footing as the other interaction mechanisms with the probability of photon survival based on the Russian roulette mechanism. The random number of the roulette wheel is compared with $F_{a}(l,\mu_{a})$ to determine whether the photon survives or annihilates at a given iteration. The survival condition of a photon is, therefore, determined by the following expression:

$$\text{survival}:\xi_{4}<F_{a}(l,\mu_{a}),$$ (8)

where $\xi_{4}$ is a random number between 0 and 1.

The probabilities of Raman scattering or fluorescence re-emission are related to the molecular structure of the biological tissue and can be modeled by their emission spectra. If the re-emission bandwidth of interest is $\left[\lambda_{a},\lambda \right]$ and the wavelength of illumination is $\lambda_{i}$, the fluorescence and Raman CDFs, which are specifying the probability of fluorescence or Raman shifts from $\lambda_{i}$ to λ, are

$$F_{F}(\lambda_{i},\lambda )=\overset{\lambda }{\underset{\lambda_{a}}{\int }}f_{F}(\lambda_{i},\lambda^{\prime })d\lambda^{\prime },$$ (9)

$$F_{R}(\lambda_{i},\lambda )=\overset{\lambda }{\underset{\lambda_{a}}{\int }}f_{R}(\lambda_{i},\lambda^{\prime })d\lambda^{\prime },$$ (10)

where $f_{F}(\lambda_{i},\lambda )$ and $f_{R}(\lambda_{i},\lambda )$ are the conversion rates of fluorescence and Raman events, which are essentially proportional to their re-emission spectra. This is because if we consider fluorescence and Raman scattering as absorption/re-emission and scattering phenomena, respectively, then the re-emission intensities in fluorescence and scattering intensity in Raman scattering are directly proportional to the corresponding fluorescence quantum yield and Raman cross section, respectively. These two intensities are, in fact, the inherent fluorescence and Raman spectra of medium under interrogation. It should be noted that in the current package, the CDFs defined in Eqs. (9) and (10) are indicated by $\rho_{F}$ and $\rho_{R}$, respectively. To decide whether, on each diffusion path, the inelastic scattering or fluorescence re-emission occurs or not, the MC package uses Eqs. (9) and (10) to calculate $\rho_{F}$ and $\rho_{R}$ for all the wavelengths of interest. Then it produces two random numbers, $\xi_{5}$ and $\xi_{6}$, and compares them with all the calculated $\rho_{F}$ and $\rho_{R}$. If the produced random numbers are smaller than the CDF value of a particular wavelength, then that wavelength will be chosen as the inelastic wavelength. If multiple wavelengths have their CDF values larger than the generated random numbers, then the shortest wavelength among them will be chosen. If the generated random numbers exceed all the CDF values of the considered wavelengths, then the diffusion would be elastic.

Finally, the reflection and refraction of photons at the interface of two layers are computed using the Fresnel coefficients. If the photon encounters an interface between two layers with refractive indices $n_{1}$ and $n_{2}$, then the package generates a random number $\xi_{7}$, between 0 and 1, and compares it with the reflectance of the interface, given by the following Fresnel relation:^18^

$$R=\frac{1}{2}[{\left(\frac{n_{1}\;\cos\;\theta_{1}-n_{2}\;\cos\;\theta_{2}}{n_{1}\;\cos\;\theta_{1}+n_{2}\;\cos\;\theta_{2}}\right)}^{2}+{\left(\frac{n_{1}\;\cos\;\theta_{2}-n_{2}\;\cos\;\theta_{1}}{n_{1}\;\cos\;\theta_{2}+n_{2}\;\cos\;\theta_{1}}\right)}^{2}],$$ (11)

where $\theta_{1}$ is the angle of incidence and $\theta_{2}$ is the angle of transmittance. In Eq. (11), R is the average reflectance for the two orthogonal polarizations of light, for in the current context of interest, we have assumed that light is unpolarized. Therefore, if $\xi_{7}$ is smaller than R, then the photon is reflected, otherwise it is transmitted.

### 2.4 Algorithm Managing Light–Tissue Interaction Events

**Fig. 1:**
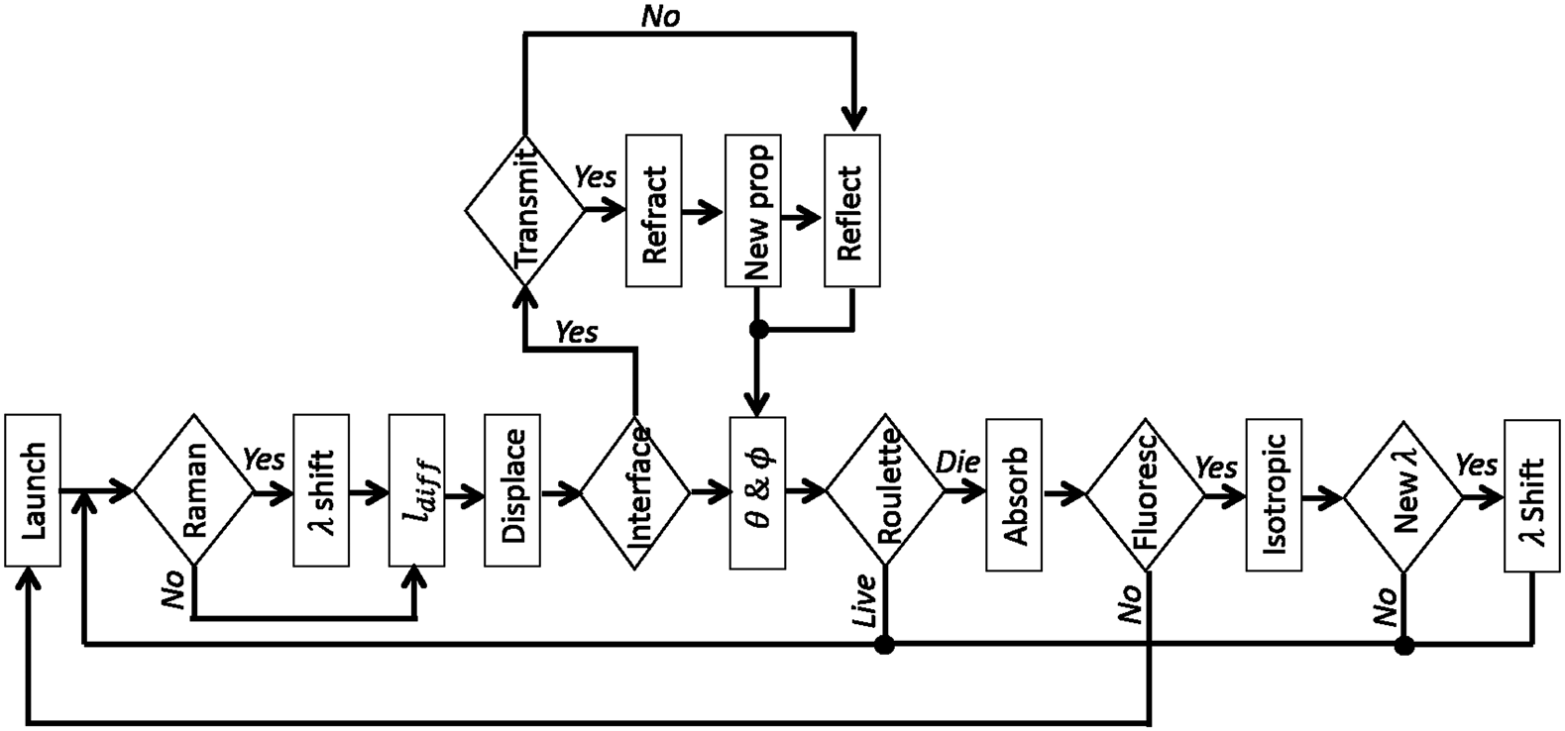
Proposed MC algorithm including all the competing events in light–tissue interaction.

Implementation details of the MC algorithm are shown in Fig. 1. To simulate elastic scattering, absorption, fluorescence, or Raman scattering, their respective physical constants are used to build pertinent PDFs and hence, sample random numbers. At the start of each step, the devised algorithm samples seven different random numbers, i.e., $\xi_{1}$ to $\xi_{7}$ as summarized in Table 1, corresponding to the length of diffusion step, azimuthal and polar directions of the diffusing photon, probabilities of absorption, florescence, and Raman scattering, and specular reflection if the next diffusion increment crosses a boundary between two media. Then, the algorithm takes the generated random numbers into account and follows survival tests for each of the above-mentioned events to decide which event dominates at that step.

**Table 1:** Generated random numbers.

Name	Variable	Corresponding equation
Diffusion free path	$l_{\mathrm{diff}}$	$l_{\mathrm{diff}}=-\ln[(1-\xi_{1})/\mu_{s}]/\mu_{s}$
Azimuthal direction of diffusion	ϕ	$\phi =2\pi \xi_{2}$
Polar direction of diffusion	θ	$\theta =\arccos\{\frac{1}{2g}[1+g^{2}-(1-\frac{g^{2}}{1-g+2g\xi_{3}}{)}^{2}]\}$
Russian Roulette	$\xi_{4}$	$\xi_{4}>1-e^{-\mu_{a}l_{\mathrm{diff}}}$
Probability of fluorescence shift	$\xi_{5}$	$\xi_{5}>\rho_{F}(\lambda )$
Probability of Raman shift	$\xi_{6}$	$\xi_{6}>\rho_{R}(\lambda )$
Probability of reflection at a surface	R	$\xi_{7}<R$

More specifically, after launching photons, the package first checks if there is any Raman shift. If there is a Raman shift, it updates the wavelength and optical properties of the medium and then computes the diffusion length [Eq. (4)] as well as azimuthal ($\phi =2\pi \xi_{2}$) and polar [Eq. (6)] directions, respectively. If there is no Raman shift, the package computes the diffusion length, azimuthal, and polar directions based on the optical properties at the wavelength of illumination. Then, the simulator checks if there is any change of interface along the diffusion segment and in the direction of the initial ϕ and θ. If there is an interface on the way, then the photon is either transmitted or reflected based on the Fresnel laws, and accordingly, the azimuthal and polar directions of subsequent diffusion (ϕ and θ) are updated. If there is no interface, then the photon travels along the path determined by $l_{\mathrm{diff}}$ and the initial ϕ and θ. At the next step, the photon undergoes the Russian roulette process and competes with absorption. If there is no absorption, the algorithm goes back to the step of Raman shift checking. If there is an absorption event, then the algorithm checks whether the photon is annihilated or undergoes fluorescence re-emission. In the case of annihilation, the package launches a new photon. In the case of fluorescence re-emission, the algorithm updates the angles ϕ and θ, as fluorescence is assumed to be an isotropic process. Furthermore, the algorithm checks if the fluorescence re-emission is at the initial wavelength or not (second last box in Fig. 1). If yes, then the package returns to the Raman shift inspection step. If no, the package updates the wavelength and optical properties for the new wavelength, and then jumps back to the Raman shift inspection step. It should be mentioned that the launched photons can repeatedly go through the described algorithm in a number of iterations set initially by the user. If before reaching the maximum number of iterations, the photon is absorbed and not re-emitted, then that whole sequence is terminated, and a new photon is generated. Furthermore, the simulation domain is considered as a void (i.e., vacuum) space where all the modeled structures are contained. Hence, the exiting photons propagate along straight lines without experiencing any scattering or absorption in the surrounding void medium. More details on the technical implementation of the MC algorithm based on OpenGL can be found in Ref. 63.

### 2.5 Physical Environment Modeling

The optical properties ($\mu_{s}$, $\mu_{a}$, g, n, $\rho_{F}$, and $\rho_{R}$) of the simulated medium need to be defined at any point in space for all the wavelengths. As is a convention in the diffuse optics literature, empirical relations for $\mu_{s}$, g, and n are used, although the code can be adapted to be compatible with the tabulated values for these constants. In the spectral therapeutic and imaging window for biological media, the dependence of the scattering coefficient on wavelength can be empirically described by a decreasing power law. Thus, the scattering is represented by an exponential equation with coefficient $a_{1}$ and power $a_{2}$

$$\mu_{s}(\lambda )=a_{1}\lambda^{-a_{2}},$$ (12)

where $a_{1}$ and $a_{2}$ are positive real numbers. The anisotropy coefficient g and refractive index n exhibit more linear behaviors

$$g(\lambda )=a_{3}+a_{4}\lambda ,$$ (13)

$$n(\lambda )=a_{5}+a_{6}\lambda .$$ (14)

The dependence of absorption, fluorescence, and Raman scattering ($\mu_{a}$, $\rho_{F}$, and $\rho_{R}$) on wavelength cannot be modeled empirically. For this reason, the package should be provided with the values of these properties at each wavelength, adapted to any desired biological application.

The MC simulation package allows flexibility in defining the illumination and detection geometries. The light sources are represented according to their position, direction, intensity (in terms of number of photons), radius, distribution (standard deviation), and emission wavelength. Thus, a monochromatic source having a Gaussian emission profile is represented only by 10 numbers. In addition, more than one source can be modeled simultaneously by adding a supplemental definition in an input file. Thus, it is possible to model a source containing several wavelengths by superimposing several sources in the file. The source radius defines the boundary beyond which no photon is emitted, which makes the simulator suitable for optical fiber sources. The cameras and sensors used in biophysics are diverse. For this reason, it is important to leave enough flexibility at the detection port. In the current version of the MC package, the pinhole camera is supported with the generic parameters tabulated in Table 2. Allowing the user to control these parameters, the simulator offers a solution adaptable to a wide variety of applications. Furthermore, in order to implement new sensors and cameras, the package allows the user to insert the corresponding camera matrix of the new sensing system and apply it to the captured photons.

**Table 2:** Generic parameters of the camera/sensor implemented in the package.

Parameter	Representation space
Position	(X,Y,Z)
Orientation	(X,Y,Z)
Resolution	(X,Y)
Field of view	(Near plane, far plane, angle of view)

## 3 Experimental Validation of the Simulation Technique

In this section, experimental measurements made in tissue phantoms using a single-point RS probe are compared with simulation results for the same geometry in synthetic phantoms in order to validate the practical utility of the new MC simulation technique.

### 3.1 Experimental Tissue Phantoms

**Fig. 2:**
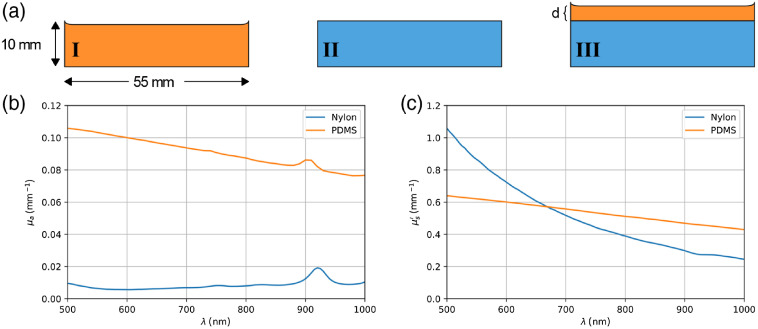
(a) Structural geometry of phantoms I (PDMS), II (nylon), and III (PDMS on the top nylon with d=913  μm). Measured (b) absorption and (c) reduced scattering coefficients of nylon and synthetized PDMS samples.

Solid tissue phantoms were fabricated from polydimethylsiloxane (PDMS) and nylon matrices. The absorption and scattering properties of the phantoms were controlled by adding India ink for absorption and titanium dioxide (TiO_2_) powder for scattering.^65^^,^^66^ PDMS and nylon were chosen in part because they have Raman signatures allowing them to be clearly distinguishable. Three phantoms were fabricated [Fig. 2(a)]: (I) a single layer of PDMS (thickness: 10 mm) with absorption and scattering agents, (II) a single layer of nylon (thickness: 10 mm) with absorption and scattering agents, and (III) a two-layer phantom made of a substrate of nylon layer (thickness: 10 mm) over which a 913-μm layer of PDMS was deposited. The thickness of the PDMS layer in phantom III was measured using a commercial optical coherence tomography (OCT) system (Thorlabs SL1310V1).^67^ The axial resolution of the system was estimated to be 20  μm in air. Two OCT B-scans were acquired by rotating the sample by 90 deg around the z axis normal to the cross section of the sample; one along x^ direction and one along y^ direction. The PDMS thickness was determined to be uniform (±10  μm) and 913-μm thick over the 3.71-mm width of the B-scans.

Characterization studies were then conducted to determine the absorption coefficient ($\mu_{a}$) and reduced scattering coefficient [$\mu_{s}^{\prime }=(1-g)\mu_{s}$] of the single-layer phantoms. Specifically, reflection and transmission spectra were acquired using a commercial PerkinElmer Lambda 1050 spectrometer equipped with a single integration sphere. The optical properties were computed using the Inverse Adding Doubling technique.^68^ Figure 2(b) shows the absorption coefficients and Fig. 2(c) shows the reduced scattering coefficients of PMDS and nylon phantoms, respectively. The optical properties are consistent with biological tissue in the NIR window. For the PDMS phantom, $\mu_{a}$ ranges from 0.11 to $0.075\;{\mathrm{mm}}^{-1}$ and $\mu_{s}^{\prime }$ varies from 0.65 to $0.45\;{\mathrm{mm}}^{-1}$ between 500 and 1000 nm. For the nylon phantom, $\mu_{a}$ changes from 0.02 to $0.01\;{\mathrm{mm}}^{-1}$ and $\mu_{s}^{\prime }$ fluctuates from 1.05 to $0.25\;{\mathrm{mm}}^{-1}$ within the wavelength range 500 to 1000 nm.

### 3.2 Experimental Raman Spectroscopy Measurements

**Fig. 3:**
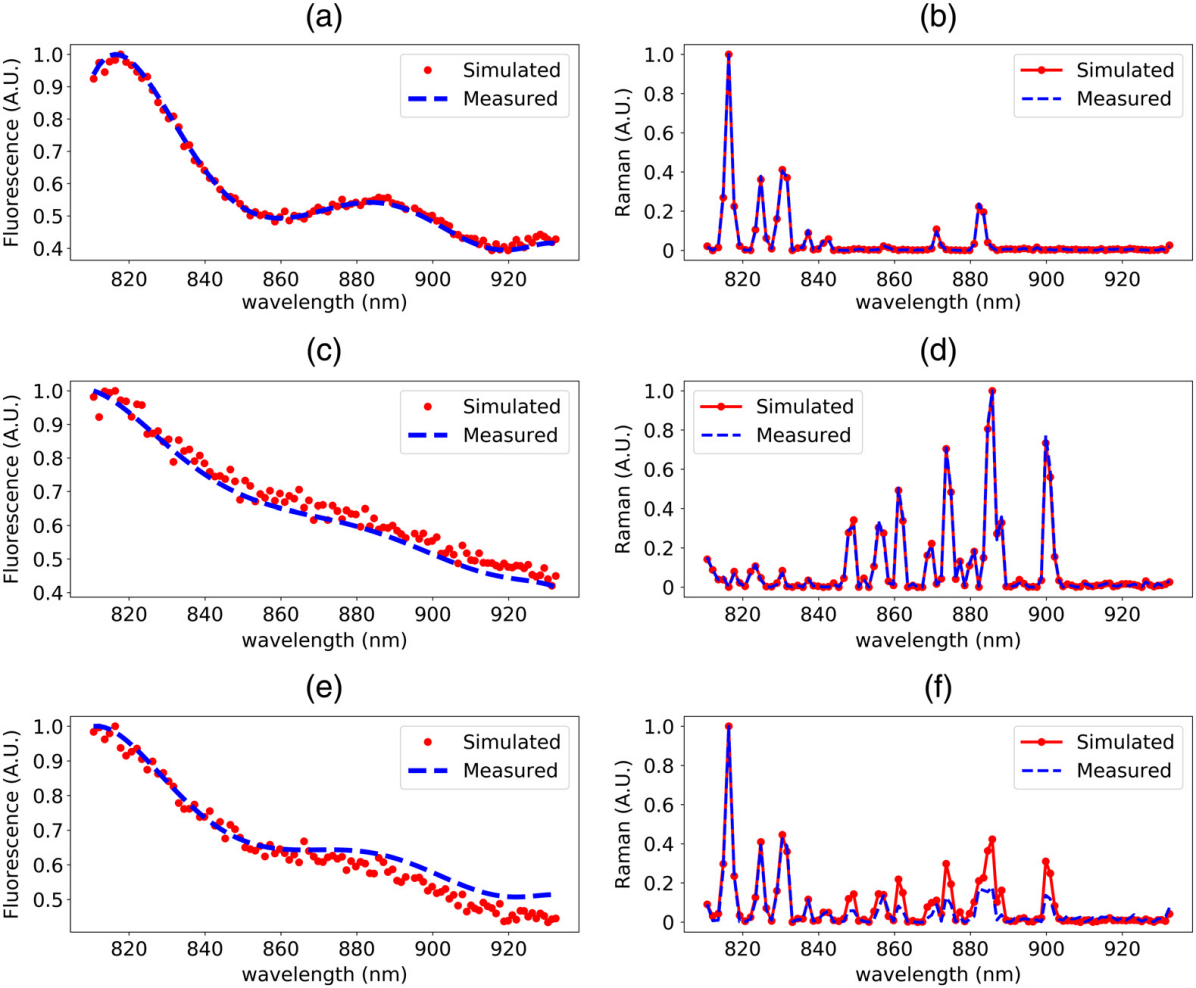
Simulated versus measured fluorescence and Raman spectra of (a), (b) phantom I, (c), (d) phantom II, and (e), (f) phantom III, respectively.

Fluorescence and Raman scattering spectra were measured with a handheld RS probe in contact with phantoms I, II, and III. All Raman acquisitions (500-μm spot size) consisted of 10 consecutive spectra acquired following excitation with a 785-nm light source (25 mW at the tip, 1-s exposure per spectrum).^6^^,^^10^^,^^69^^,^^70^ The raw signals were processed to isolate the contribution associated with the Raman effect: (1) the background signal (measurement acquired with laser turned off) was subtracted and the resulting spectrum corrected for system response by normalizing it to a measurement made on a Raman standard (SRM2241; NIST, Gaithersburg, Maryland);^71^ (2) the remaining low-frequency background (mainly fluorescence) was removed using the rolling ball algorithm;^72^ and (3) the spectrum was normalized with standard normal variate (SNV) technique to have a mean of zero and standard deviation of one. Figure 3 shows the post-processed spectra for each phantom. All expected Raman peaks for PDMS and nylon were detected as seen in Figs. 3(b) and 3(d), respectively. The measured Raman spectrum for the two-layer phantom [Fig. 3(f)] shows all peaks associated with the top PDMS layer and several peaks associated with nylon in the higher wavelength range above 800 nm.

### 3.3 Monte Carlo Simulations

**Fig. 4:**
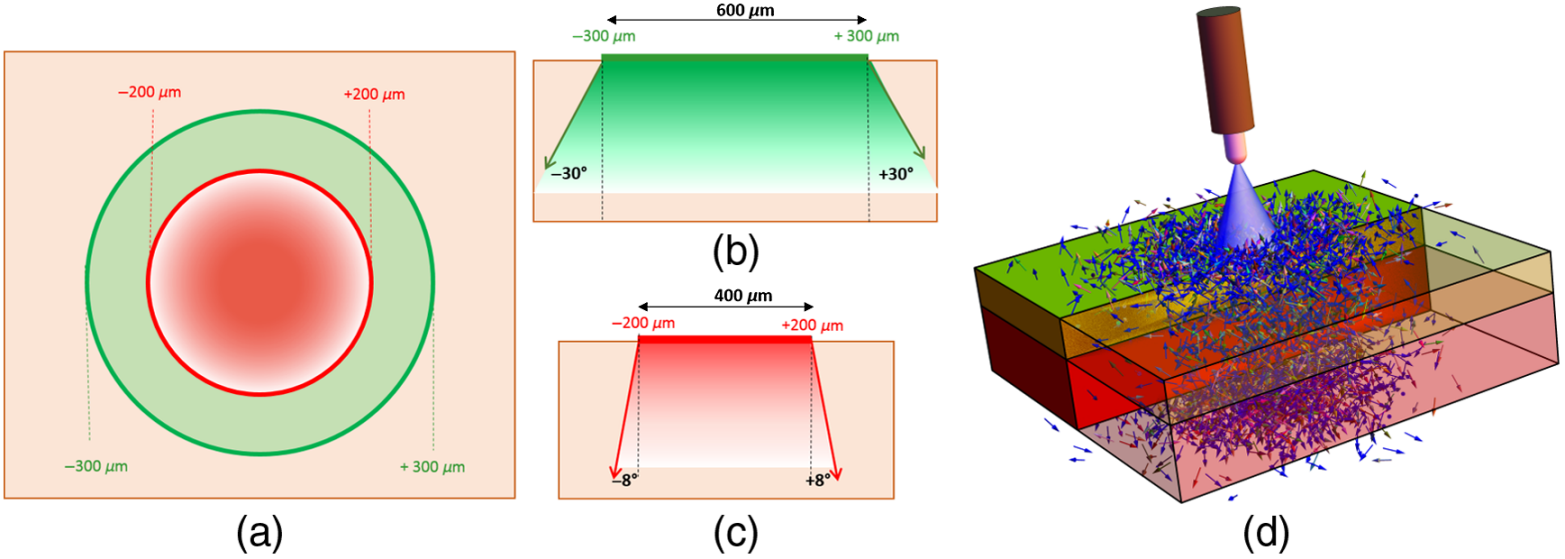
(a) Cross-sectional view of the handheld interrogating probe. The red region with a diameter of 400  μm indicates the source port, while the green region with a diameter of 600  μm illustrates the collecting port. (b) Side-view of the detector geometry. (c) Side-view of source geometry. (d) Elastic and inelastic photon diffusion in a two-layered sample under illumination of a light beam.

An MC light transport protocol was developed [Fig. 4(d)] to run simulations consistent with the illumination/detection geometry of the handheld single-point RS probe as well as with the geometry and optical properties of phantoms I, II, and III (Fig. 2). Figures 4(a)–4(c) show the cross-sectional and side views of the simulated source and detector configurations. The detection area was approximated by a cylinder of 600-μm diameter surrounding the illumination area modeled by a 400-μm diameter disk. The acceptance angles (numerical apertures) for illumination and detection were 8 deg and 30 deg, respectively.

Three-dimensional (3-D) grids of voxels were generated to emulate the geometry of the phantoms: 256×256×256 for the single-layer phantoms I and II and 501×501×203 for the two-layer phantom III. Optical properties parameters were assigned to each voxel to match conditions met in the experimental tissue phantoms. Those included a refractive index of n=1.4 and the measured absorption and reduced scattering coefficients reported in Fig. 2. The measured background (the residual after applying the rolling ball algorithm) and resulting Raman spectra of the single-layer PDMS and nylon were used to assign relative probabilities at each voxel associated with the fluorescence and Raman scattering processes, respectively. As noted while presenting Eq. (10), the fluorescence and Raman conversion probabilities are proportional to their corresponding spectra of re-emission and scattering, respectively. Accordingly, the acquired spectra from single layer PDMS and nylon were multiplied by a proportionality constant and used as probability conversion rates ($\rho_{F}$ and $\rho_{R}$) in the CDF manner at 100 discrete wavelengths equally distributed between 810 and 932 nm. Tissue excitation was modeled by a monochromatic point source with an excitation wavelength of 785 nm. All simulations were performed on a DELL Precision 7920 Tower workstation with an 8 core Intel Xeon Silver 4110 microprocessor (2.1 GHz, 3.0 GHz Turbo), an NVIDIA Quadro P4000 graphic card, and OpenGL 4.5. As illustrated in Fig. 4(d), a total of $7.2\times 10^{8}$ photons in 3600 batches were launched onto the center of the top face for each phantom to simulate Raman scattering and fluorescence re-emission at 100 discrete wavelengths equally spaced between 810 and 932 nm. The required time for the MC simulating and subsequent post-processing calculations were 5.5 and 4 s per batch, respectively. The source was approximated as a Gaussian distribution with standard deviation of 0.1  μm. A post-processing analysis was applied at the detection end to count the Raman photons collected within the aperture of the detectors.

The simulations for each phantom resulted in spectra that were post-processed using the same background removal technique as for the experimental spectra. Figures 3(a), 3(b) and 3(c), 3(d) show the SNV-normalized experimental and simulated spectra for the pure PDMS and nylon phantoms, respectively. The post-processed simulated and experimental spectra are in agreement, preliminarily demonstrating the efficacy of the method to simulate inelastic Raman scattering in a diffusive medium. Figures 3(e) and 3(f) show the simulated and experimental results for the two-layer phantom, respectively. The two spectra show overall good agreement with minor differences between the measured and simulated curves at longer wavenumber shifts, where most Raman nylon peaks of the nylon substrate are located.

## 4 Inelastic Scattering Sampling Depth

### 4.1 Simulation Geometry and Depth Sensing Evaluation Metrics

A comprehensive study of the effects of absorption, elastic, and inelastic scattering on the depth of Raman conversion was conducted. Because of light diffusion in tissue, each optical measurement was associated with a distribution of photons having sampled different sensing depths. From the Beer–Lambert law, the fraction of detected photons having sampled a given depth d exponentially decreases with depth. In the analysis presented here, Raman sensing depth is defined as the depth after which a specific cumulative percentage of detected Raman photons was scattered. To ensure that the detected signals represented a realistic sensing depth, two values were computed as references. Specifically, depth sensing values were computed associated with the depths after which 75% or 90% of all Raman photons were generated in the face of all other competing interaction mechanisms.

To conduct the Raman depth sampling analysis, a series of MC simulations were done. In each simulation, the *in silico* phantom was first assumed to be a semi-infinite space (i.e., z<0). The illuminating source was modeled by a monochromatic point source (λ=785  nm), which had a Gaussian radial distribution with a standard deviation of 0.1  μm and was located at the center of the top face of the phantom. However, to minimize computational time and required memory, the simulated phantom was approximated as a slab of $2\times 2\times 2\;{\mathrm{cm}}^{3}$, which was modeled by a 3-D grid composed of 256×256×256 voxels. For each simulation, $6\times 10^{7}\text{ photons}$ in 300 batches were launched, where the required time for the MC simulation and subsequent post-processing analysis were 5.5 and 4 s per batch, respectively. As for the collection part, a post-processing analysis was performed to count the Raman photons collected by an aperture identical to the detector in Fig. 4.

### 4.2 Impact of Inelastic Scattering Conversion Rate on Raman Depth Sampling

**Fig. 5:**
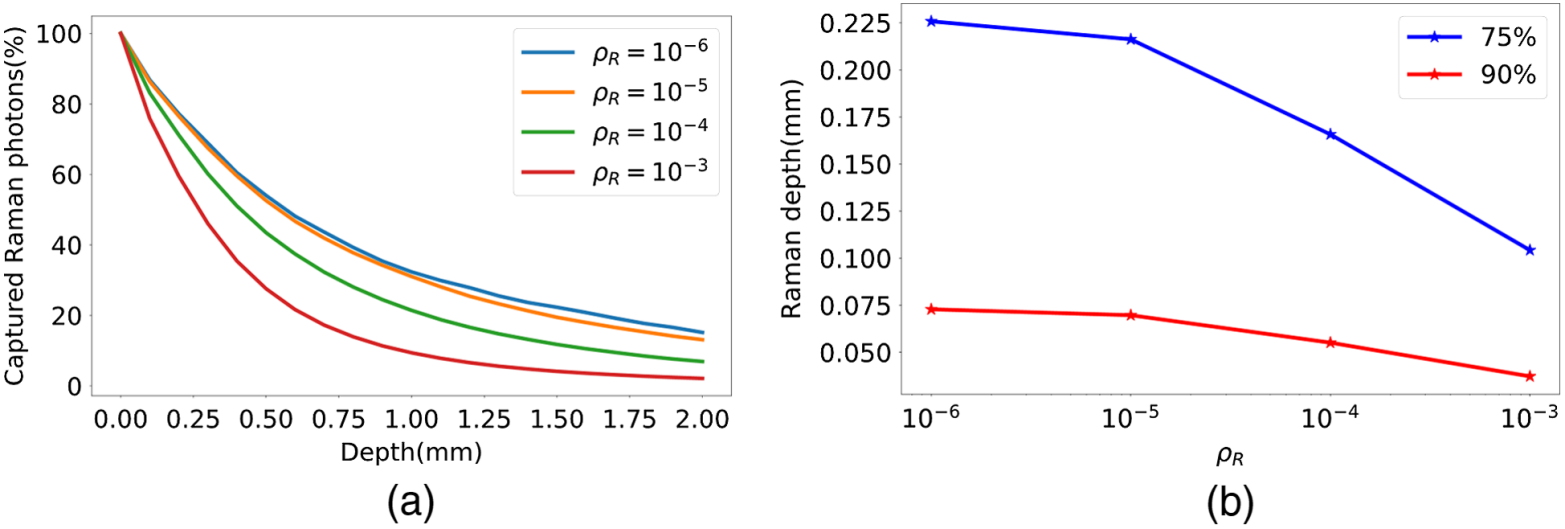
(a) Cumulative percentage of detected Raman photon versus penetration depth for various Raman conversion rates within a phantom with $\mu_{s}^{\prime }=1\;{\mathrm{mm}}^{-1}$ and $\mu_{a}=0.001\;{\mathrm{mm}}^{-1}$. (b) 75% and 90% Raman depths versus conversion rate.

The effect of the Raman conversion rate $\rho_{R}$ on the Raman depth was first studied. Figure 5(a) shows the cumulative percentage of detected Raman photons versus the Raman penetration depth for a phantom with $\mu_{s}^{\prime }=1\;{\mathrm{mm}}^{-1}$ and $\mu_{a}=0.001{\mathrm{mm}}^{-1}$, and Raman conversion rate $\rho_{R}$ varying between $10^{-6}$ and $10^{-3}$. As observed in this figure, increasing the Raman conversion rate increases the slopes of the curves, which indicates that the Raman depth is approximately inversely proportional to the conversion rate. This relation is more clearly visible in Fig. 5(b), where the 75% and 90% Raman depths are plotted for various conversion rate values. It is observed that as the Raman conversion rate increases, most of the propagating photons undergo a Raman shift within shallower layers.

### 4.3 Impact of Absorption and Elastic Scattering on Raman Depth Sampling

**Fig. 6:**
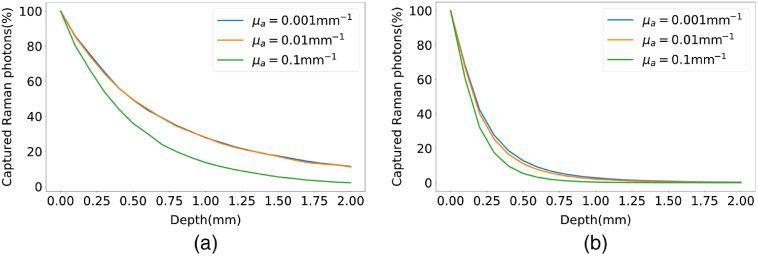
Cumulative percentage of detected photons versus penetration depth for various absorption coefficients of a phantom with $\rho_{R}=10^{-5}$ and (a) $\mu_{s}^{\prime }=1\;{\mathrm{mm}}^{-1}$, (b) $\mu_{s}^{\prime }=10\;{\mathrm{mm}}^{-1}$.

**Fig. 7:**
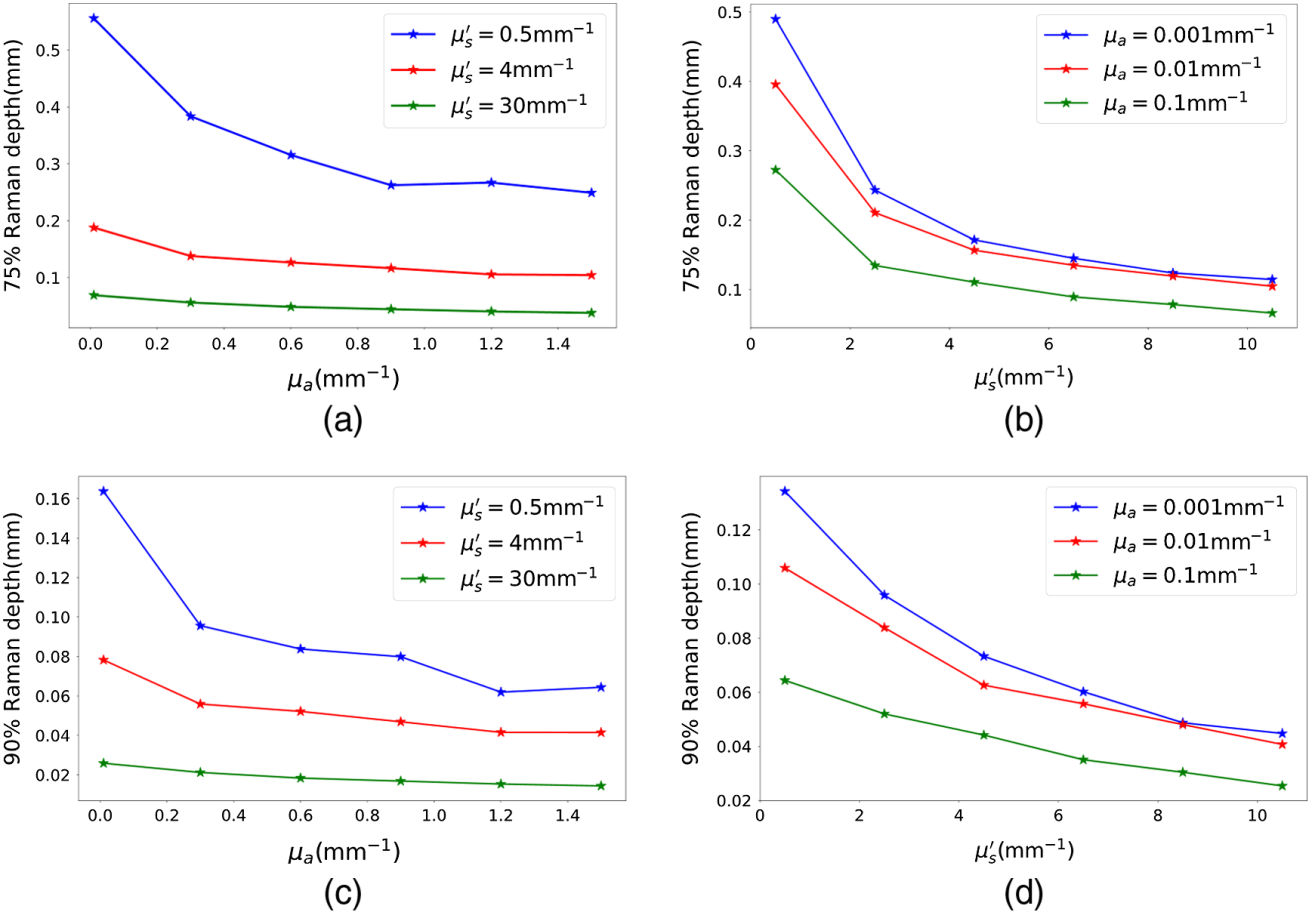
(a) and (c) 75% and 90% Raman depth versus absorption coefficient for various $\mu_{s}^{\prime }$ and $\rho_{R}=10^{-6}$. (b) and (d) 75% and 90% Raman depths versus scattering coefficient for various $\mu_{a}$ and $\rho_{R}=10^{-6}$.

The effects of absorption and elastic scattering on Raman sensing depth are illustrated in Figs. 6 and 7. Figure 6(a) shows the photon intensity curves for $\mu_{s}^{\prime }=1\;{\mathrm{mm}}^{-1}$ and various absorption coefficients. In this figure, it is shown that for a highly absorptive medium ($\mu_{a}=0.1\;{\mathrm{mm}}^{-1}$), the slope of the cumulative captured photon intensity curve is larger compared to those with lower absorption coefficients, indicating shallower Raman sensing depths for more absorptive media. Figure 6(b) shows the photon intensity curves for a higher scattering coefficient of $10\;{\mathrm{mm}}^{-1}$ and varying absorption coefficients. For the higher scattering coefficient, all the intensity curves become much sharper, indicating much shallower Raman sensing depths for more scattering media. This indicates the dominance of elastic scattering event on the depth of Raman re-emission. These two facts are more visible in Fig. 7, where the 75% and 90% Raman sensing depths are traced versus the reduced scattering and absorption coefficients, respectively. Comparing Figs. 7(a), 7(c) and 7(b), 7(d) reveals that the effect of absorption coefficient on the Raman sensing depth is less significant than that of the scattering coefficient. According to these figures, for a constant $\mu_{s}^{\prime }$ value, increasing $\mu_{a}$ results in a smaller decrease in the 75% and 90% Raman sensing depth compared to the depth decrease observed by increasing $\mu_{s}^{\prime }$ for a fixed $\mu_{a}$ value. Furthermore, as $\mu_{s}^{\prime }$ grows, the 75% and 90% Raman depth curves versus $\mu_{a}$ become flatter, which indicates that the Raman sensing depth is less influenced by the absorption in media with stronger scattering coefficients. In other words, for higher amounts of $\mu_{s}^{\prime }$, the Raman sensing depth curves versus $\mu_{a}$ behave like parallel lines, which shows that for stronger scattering coefficients, the amount of change in the sensing depth is progressively independent of $\mu_{a}$. Finally, as can be seen in Figs. 7(a) and 7(c), the blue curves are not following the general expected behavior at the last two points, where kinks appear. This is due to the fact that, at such points, the absorption coefficient is much larger than the reduced scattering coefficient, and hence, estimating the photon paths and fluence rate is out of the realm of the diffusion equation. Nevertheless, such extreme values for absorption and reduced scattering coefficients are rarely seen in biological tissues.

## 5 Discussion

Prior to being used in human subjects, optical imaging instruments should undergo an iterative design procedure facilitated by tissue phantoms with realistic geometrical and optical properties such that a reliable assessment of the imaging performance and utility can be obtained. In this regard, the optimized system-specific parameters can be estimated by optical simulation of the designed imaging instrument, e.g., by detailed ray tracing analysis of included optical components as well as their arrangements. Tissue-specific technical features are usually estimated based on tissue phantom experiments,^73^ which model a range of optical properties representing the expected inter- and intrapatient heterogeneity, potential impact of specular reflections, and possible variations in tissue surface characteristics including curvature and texture. However, in order to complement the phantom analysis and estimate a wider spectrum of optical properties of the tissue under interrogation, in diffuse optics, numerical light transport simulations have been invoked in a number of modern clinical applications. A few examples of such numerical studies are fluorescence imaging systems for surgical guidance,^74^ source–sensor geometries in diffuse optics applications,^75^ and diffuse optical tomography systems.^76^

This article presented the development of a spectroscopic MC code to simulate both elastic and inelastic light interactions in biological tissue. The Raman and fluorescence spectroscopy capabilities of the MC package were validated showing agreement between simulations and experimental measurement in pure materials, here either nylon or PDMS tissue phantoms. In layered materials (a thin layer of PDMS over a substrate of nylon), differences were observed between the measured and simulated spectra in the higher wavelength regime, where most of the nylon Raman peaks are located. This could potentially be due to multiple factors including the inability of the reduced scattering approximation formula incorporated in the package (Mie scattering) to effectively model scattering at larger depths, the inaccuracy in estimating the refractive index mismatch between the two layers, and/or inefficiencies of the normalization process applied to extract the Raman and fluorescence spectra.

To illustrate the analytic capabilities of the MC package, a detailed analysis was performed to estimate the depth sensing capabilities of an existing surgical-guidance handheld probe instrument. The study was performed with the objective of quantifying the impact of tissue optical properties (absorption and elastic scattering) and the relative probability of occurrence of the Raman effect on inelastic scattering depth sampling. Although realistic values for biological tissue parameters such as absorption and elastic scattering can be found in the literature, no realistic values were available to estimate the relative probability, or physical cross section, of inelastic scattering (herein referred to as Raman conversion rate) in biological tissue. As a result, values associated with different orders of magnitude for this parameter were simulated to evaluate its impact on depth sampling (Table 3).

**Table 3:** Optical properties used in the sensing analysis and corresponding calculated Raman sensing depths.

Parameters				Depth of 75% sensitivity (μm)	Depth of 90% sensitivity (μm)
Absorbtion $\mu_{a}$ (${\mathrm{mm}}^{-1}$)	Scattering $\mu_{s}^{\prime }$ (${\mathrm{mm}}^{-1}$)	Mean free path (μm)	$P_{\text{Raman}}$		
0 to 1.4	0.5	200	$10^{-6}$	270 to 600	75 to 160
	4	25		140 to 190	41 to 80
	30	3.3		10 to 80	15 to 25
0.001	1	100	$10^{-3}$ to $10^{-6}$	105 to 225	40 to 75

It was shown that in media with higher inelastic cross-section values, the rate of Raman conversion at superficial layers is higher and the sensing depth associated with the probing instrument decreases accordingly. This is expected because when the Raman cross section increases, the likelihood of Raman conversion of photons as they diffuse across shallow layers grows, which results in a higher percentage of superficial Raman scattering events. Since the probability of two consecutive Raman shifts for a single photon is negligible, the likelihood of another Raman scattering at deeper layers for the already-Raman-shifted photons becomes vanishingly low, and hence, most of the detected Raman photons are those re-emitted from upper layers. However, for lower conversion rates, the overall intensity of the collected Raman signal is also lower. Therefore, reducing the Raman conversion rate for the sake of increasing the Raman depth is at the expense of lowering the count associated with inelastically scattered photons. As a result, there is a trade-off between Raman depth sensing and Raman SNR. It was also shown that absorption has a direct impact on Raman sensing depth: larger tissue absorption values lead to smaller sensing depths. This makes sense intuitively, since for smaller absorption coefficient values the likelihood that photons diffuse over longer distances increases, hence, a larger relative fraction of elastic scattering events occurs.

In the course of photon transport within tissue, among the four competing events, elastic scattering acts as a stimulator for other three events. In other words, the higher the elastic scattering coefficient, the shorter would be the diffusion length between two consecutive scattering events, the greater would be the number of collisions between photons and molecules, and therefore, the higher would be the chance of absorption, fluorescence, and Raman scattering. In a more quantitative description, in a medium with a large scattering coefficient, the spatial variation of electric polarizability dα/dx, which mainly contributes to the amplitude of re-emitted Raman signal, is large. Therefore, a positive incremental addition to the scattering coefficient, such as $\Delta \mu_{s}^{\prime }$, leads to a positive incremental increase $\Delta \rho_{R}$ in the Raman conversion rate, i.e., $\mu_{s}^{\prime }+\Delta \mu_{s}^{\prime }\to \rho_{R}+\Delta \rho_{R}$. Hence, as discussed in the previous section, as the scattering coefficient becomes larger, the Raman conversion rate increases, and the event of Raman shift at upper layers becomes more likely. Therefore, the Raman sensing depth in tissues or phantoms with higher scattering coefficients is less than the depth in structures with lower scattering coefficients.

As a final point, it should be mentioned that the discussed MC algorithm is a direct method of light transport calculation, which may require large number of photons, especially in situations where one of the competing processes has a very small probability to trigger this. As reviewed comprehensively in Ref. 21, there are several options to accelerate the MC modeling of light transport in turbid media, including scaling, perturbation, and convolution. In these accelerated techniques, the MC analysis is performed indirectly by modifying an existing base MC calculation. As noted earlier, the developed package gives the option of recording the final location, direction, and wavelength of the simulated photons. Hence, in principle, a user can readily use those recorded data as a base simulation and perform accelerated MC analysis in new cases. Following the given explanations and shown results, it is hoped that the developed package will bring more understanding in the area of light transport modeling and equip the contributing research community with a versatile toolset for optical detection and treatment as well as surgical guidance.
